# Supplementary material for: In Vitro Analysis of Matched Isolates from Localized and Disseminated Gonococcal Infections Suggests That Opa Expression Impacts Clinical Outcome
Source: Pathogens. 2022 Feb 7;11(2):217. doi: 10.3390/pathogens11020217 (PMC8880309; doi:10.3390/pathogens11020217)
Supplement: Supplementary file 1 [file pathogens-11-00217-s001.zip › Table S1 MLST allele of transmigration related genes in clinical isolated Neisseria gonorrhoeae from differen.pdf]

Table S1: MLST allele of transmigration related genes in clinical isolated *Neisseria gonorrhoeae* from different anatomical locations of the patients

Patient	21		23		29		61		63	
Locus	Blood	Cervix	Blood	Cervix	Blood	Urethra	Blood	Cervix	Blood	Cervix
NEIS2020 (PorB)	582 (PIA)	582 (PIA)	957 (PIA)	957 (PIA)	957 (PIA)	957 (PIA)	957 (PIA)	1620 (PIB)	582 (PIA)	582 (PIA)
NEIS2109 (nhba)	89	89	679	679	73	73	73	89	653	653
NEIS0210 (pilE)	1858	1866	474	1895	1895	1897	1843	550	935	935

*Allele numbers are generated from PubMLST.org
